# Use of metoclopramide in the first trimester and risk of major congenital malformations: A systematic review and meta-analysis

**DOI:** 10.1371/journal.pone.0257584

**Published:** 2021-09-20

**Authors:** Li Sun, Yang Xi, Xiaoke Wen, Wei Zou

**Affiliations:** 1 Department of Pharmacy, Hunan Provincial Maternal and Child Health Care Hospital, Changsha, Hunan, China; 2 Department of Pharmacy, the Second Xiangya Hospital, Central South University, Changsha, Hunan, China; 3 Institute of Clinical Pharmacy, Central South University, Changsha, Hunan, China; University of Oslo, NORWAY

## Abstract

**Background:**

Nausea and vomiting of pregnancy affects up to 80% of pregnant women, it typically occurs during the first trimester which is the most sensitive time for environmental exposures given organogenesis. Metoclopramide is an antiemetic drug used widely during NVP, but the findings of studies evaluating its safety of use in pregnancy is inconsistent. Therefore, we conducted a systematic review and meta-analysis to assess whether metoclopramide use during first trimester of pregnancy is associated with the risk of major congenital malformations.

**Methods:**

The systematic search using database included Pubmed, Embase, Web of science, and Cochrane library. Studies written in English, comprising with an exposed group and a control group, reporting major congenital malformation as an outcome were included.

**Results:**

Six studies assessing a total number of 33374 metoclopramide-exposed and 373498 controls infants were included in this meta-analysis. No significant increase in the rate of major congenital malformation was detected following metoclopramide use during first trimester (OR, 1.14; 95% CI, 0.93–1.38).

**Conclusions:**

Metoclopramide use during first trimester of pregnancy was not associated with the risk of major congenital malformations.

## 1. Introduction

Nausea and vomiting of pregnancy (NVP) is a common pregnancy-related medical condition that typically occur during the first trimester, affecting about 80% pregnant women [1]. Recurrence of NVP with subsequent pregnancies ranges from 15–81% [2]. The most severe form of NVP, hyperemesis gravidarum (HG), characterized with intractable nausea and vomiting, often necessitated hospitalization by leading to weight loss, electrolyte imbalance, dehydration, nutritional deficiencies, ketonuria, even forced termination of pregnancy. The American College of Obstetricians and Gynecologists (ACOG) and “Obstetrics Subgroup, Chinese Society of Obstetrics and Gynecology, Chinese Medical Association” both recommended vitamin B6 (pyridoxine) alone or it combined with doxylamine as the first-line pharmacotherapy for NVP treatment, which is considered safe and effective [3,4], but a lot of countries do not have doxylamine (such as China and Germany). Metoclopramide is a dopamine receptor antagonist, which works via blocking the dopamine receptor in the chemoreceptor trigger zone and depressing the vomiting center in the brain [5]. It is widely used in NVP at the situation that a treatment with vitamin B6 or antihistamine has failed. Since the first trimester is a sensitive period for fetal teratogenesis, the safety of medication in this period has attracted much attention. Arvela et al found that metoclopramide crosses the placenta rapidly at term, and it was detectable in all the umbilical arterial, venous as well as amniotic fluid samples [6]. However, the extent of placental transfer of metoclopramide during the other stages of pregnancy is unknown. Metoclopramide has been widely used for treating NVP, so that even a small excess risk for adverse pregnancy outcome would make a significant clinical implication. In recent, there are some studies evaluating the outcome of metoclopramide use in pregnancy have inconsistent findings [7,8]. Therefore, we performed a meta-analysis to provide a comprehensive assessment of the association between metoclopramide use in first trimester and major congenital malformations. To the best of our knowledge, this is the first meta-analysis exploring the risk of major congenital malformations and metoclopramide use in the first trimester.

## 2 Materials and methods

### 2.1 Search strategy

We followed the Preferred Reporting Items for Systematic Reviews and Meta-Analyses (PRISMA) [9] guidelines to perform and report our meta-analysis. Searches were conducted by the study authors in Pubmed, Embase, Web of science, and Cochrane library from inception to June 2021. Combinations of MeSH and text words in our search string in Pubmed. the Embase, Web of science, and Cochrane library were searched with similar search strings. The search string used was Metoclopramide AND (pregnancy OR pregnant OR “congenital abnormalities” OR abnormalities OR Deformity OR “birth defect” OR defect OR “congenital defects” OR deformity OR malformation OR teratogen). References of selected articles were also hand searched to ensure all possible articles were captured. Two independent reviewers performed article selections, and disagreements were resolved through consensus.

### 2.2 Inclusion and exclusion criteria

All available RCTs, cohort, and case-control studies were selected. A study was considered eligible if it met the following criteria: 1) Written in English; 2) Reporting human data; 3) Exposure to metoclopramide during the first trimester; 4) Control groups should not be exposed to metoclopramide; 5) Reporting major congenital malformation as an outcome; 6) The data reported were not overlapping with another study. The exclusion criteria were case reports, conference abstracts, series, editorials, commentaries, reviews, and articles in other languages than English.

### 2.3 Quality assessment

Two independent authors using the Newcastle-Ottawa Scale (NOS) system to assessed the quality of each included study [10], in which a study would be evaluated with “star system” on three broad perspectives: 1) The selection of the study groups; 2) The comparability of the groups; 3) The ascertainment of either the exposure or outcome of interest for case–control or cohort studies, respectively. Each study can be awarded stars at the maximum number of nine, and if a study earning 6 or higher amounts of stars were regarded as high-quality one [11].

### 2.4 Outcome measures

The outcome of interest for this meta-analysis was overall major congenital malformations.

Congenital malformations can be defined as abnormalities of body structure or function that are present at birth and are of prenatal origin. Major congenital malformations are defined as structural changes that have significant medical, social or cosmetic consequences for the affected individual, and typically require medical intervention. In contrast, minor congenital malformations, although more prevalent among the population, are structural changes that pose no significant health problem in the neonatal period and tend to have limited social or cosmetic consequences for the affected individual [12].

### 2.5 Data extraction

Two authors conducted study selection and data collection independently. The following baseline data were collected for each study: first author, publication year, study design, country, data source, study period, inclusion criteria, control, period of pregnancy of drug use, dosage of metoclopramide, method of congenital malformation diagnosis, malformations in women exposed to metoclopramide, malformations in women not exposed to metoclopramide, results relevant to this meta-analysis, quality assessment (Newcastle-Ottawa scale). Any disagreement was resolved by discussion.

### 2.6 Meta-analytic methods

Statistical analysis was performed by using software Review Manager version 5.3 (RevMan, The Cochrane Collaboration, Oxford, UK) and Stata version 14.0 (Stata Corporation, College Station, TX). Given the outcomes were dichotomous, we choose the Mantel Haenszel method for analyzing the outcomes in our meta-analysis. The pooled outcomes were calculated using the odds ratio (OR) with 95% confidence intervals (CI). The degree of heterogeneity between studies was determined by Q-statistic and inconsistency index (*I*^2^). Depending on the heterogeneity, meta-analyses were conducted using either a fixed-effect (FE) model or random-effect (RE) model. Lack of heterogeneity was defined as *P* > 0.10 and *I*^2^ < 50%. The FE model was used when there was no significant heterogeneity between studies. In the other case, the RE model were used to combine the results [13]. Publication bias was assessed by Begg’s test [14] and Egger test [15]. Publication bias was considered when the *P*- value was < 0.05.

## 3. Results

### 3.1 Study selection

**Fig 1:**
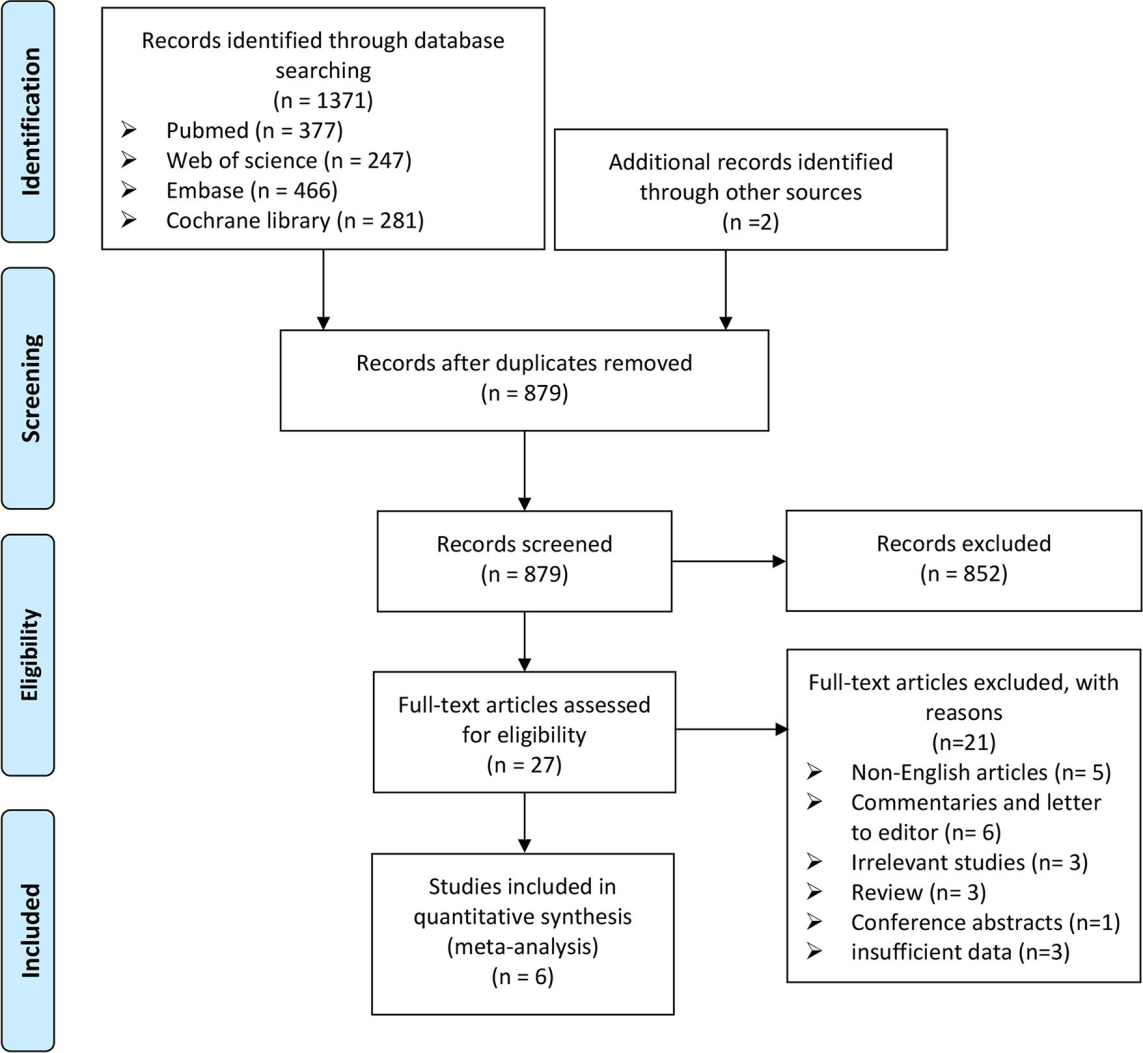
PRISMA flow diagram of study selection process in this meta-analysis.

There are 1371 references collected in the databases according to the search strategy described in the method (Pubmed = 377, Web of science = 247, Embase = 466, Cochrane library = 281). Two additional references were identified from handsearching [16,17]. Of these, 879 citations remained after duplicates were removed. After title and abstract screening, 27 abstracts were selected for full text review and 6 studies were included in this meta-analysis [7,8,18–21]. Fig 1 shows the PRISMA flow diagram of the study selection process.

### 3.2 Study characteristics

All 6 included studies were published between 2002 and 2021. retrospective cohort studies, prospective cohort, observational cohort, and register-based cohort studies were included in this meta-analysis. Six different countries contributed with data, with two studies from Denmark, one from Israel, Canada and Japan, and one international collaborations (Israel, Italy, Brazil and Canada). All included studies investigated the risk of major congenital malformation in relation with first-trimester exposure to metoclopramide. A summary of the included studies can be seen in Table 1.

**Table 1:** Characteristics of included studies in this meta-analysis.

First author/Publication year	Berkovitch et al 2002 [18]	Matok et al 2009 [19]	Pasternak et al 2013 [7]	Berard et al 2019 [8]	Hishinuma et al 2021 [21]	Sakran et al 2021 [20]
Study design	Prospective cohort	Retrospective cohort	Register-based cohort	Prospective cohort	Observational cohort	Prospective cohort
Country/Data source	Six teratogen information centers (one each in Italy and Brazil and Canada and three in Israel)	Israel Clalit Health Services Soroka Medical Center	Denmark Medical Birth Register National Patient Register	Canada Quebec Pregnancy Cohort	Japan 1.Clinic for “Pregnancy and Medicine” of Toranomon Hospital. 2.the Japan Drug Information Institute in Pregnancy of National Center for Child Health and Development	Israel Israeli Teratology Information Service (TIS)
Study period	N/A	1998.1.1–2007.3.31	1997.1.1–2011.3.31	1998.1–2015.12	1988.4–2017.12	2010–2014
Inclusion criteria	The women who called 1 of 6 teratogen information services to obtain information about the potential risks of metoclopramide use during pregnancy.	All women 15 to 49 years of age who were registered in Clalit Health Services and were living in the Beer-Sheva district and who had a singleton delivery at Soroka Medical Center	All pregnancies in Denmark with delivery dates or dates of abortive outcome January 1, 1997, through March 31, 2011.	1. continuous prescription drug insurance coverage of at least 12 months before the 1DG and during pregnancy and 2. pregnancies with a liveborn singleton, given that multiplicity is associated with MCMs	The women who consulted the Toranomon Hospital or National Center for Child Health and Development regarding the safety of drugs during pregnancy.	Pregnant women counseled by the TIS in regarded to ondansetron in the first trimester. The pregnant women with NVP treated with metoclopramide in the first trimester was comparison groups.
Control	The women who were counseled at the participating centers during the study period for the use of drugs that are known to be nonteratogenic and nonembryotoxic.	All women who did not take metoclopramide during the first trimester over the course of the study period.	Did not use metoclopramide throughout the respective exposure time window.	Did not exposure to any antiemetics during the same time window	The pregnant women who took control drugs (considered to be nonteratogenic) in the first trimester.	Pregnant women counseled for nonteratogenic exposure at a ratio of 1:4 in a similar time frame.
Period of pregnancy of drug use	Period between the 4th and 13th week of gestation	The first trimester of pregnancy (at 13 weeks’ gestation or earlier)	The first trimester (pregnancy start through 12 gestational weeks)	The first trimester (first day of the last menstrual period 98 days of gestation)	The first trimester (4th week to 13th week of pregnancy)	The first trimester
Dosage of duration metoclopramide	The mean daily dose of metoclopramide (126 women out of 175) was 23 ± 10 (10 to 40) mg and the duration of therapy was 10 ± 10 (1 to 35) days.	The mean (±SD) exposure to metoclopramide during the first trimester was 7.2±5.4 defined daily doses.	N/A	the mean duration for metoclopramide use was 17.7 days	N/A	N/A
Method of congenital malformation diagnosis	as defined by Marden et al [22]	The Metropolitan Atlanta Congenital Defects Program of the Centers for Disease Control and Prevention (CDC)	EUROCAT (European Surveillance of Congenital Anomalies) classification	ICD-9 and ICD-10 codes	EUROCAT (European Surveillance of Congenital Anomalies	Guidelines for case classification publish by Rasmussen et al [23].
Malformations in Women Exposed To Metoclopramide	7/158	major: 182/3458 minor: 133/3458de	721/28486	103/939	8/224	1/109
Malformations in Women Not Exposed to Metoclopramide	8/164	major: 3834/78245 minor: 2730/78245	3024/113698	14402/179106	27/1554	8/731
Results relevant to this meta-analysis	**Use of metoclopramide during the first trimester** Major malformation: RR: 0.91 (0.34–2.45)	**Use of metoclopramide during the first trimester** Major congenital malformations: aOR: 1.04 (0.89–1.21) Minor congenital malformations: aOR: 1.10 (0.92–1.31)	**Use of metoclopramide during the first trimester** Major malformations overall: aOR: 0.93 (0.86–1.02)	**Use of metoclopramide during the first trimester** Major congenital malformations aOR: 1.27 (1.03–1.57) Genital organ defects aOR: 2.26 (1.14–4.48)	**Use of domperidone during the first trimester** Major malformation: aOR:1.86(0.73–4.70) **Use of metoclopramide during the first trimester** Major malformation: aOR:2.20(0.69–6.98)	**The overall rate of major anomalies did not significantly differ between the groups** [4/200 = 2.0% (ondansetron), 1/109 = 0.9% (metoclopramide), and 13/731 = 1.8% (NTE)].
Quality assessment (Newcastle-Ottawa scale)	-***/**/***8	***-/**/*—6	***-/**/***8	-***/**/***8	-***/**/***8	-***/**/***-7

### 3.3 Meta-analysis of major congenital malformation rates in metoclopramide-exposed and non-exposed (main results)

**Fig 2:**
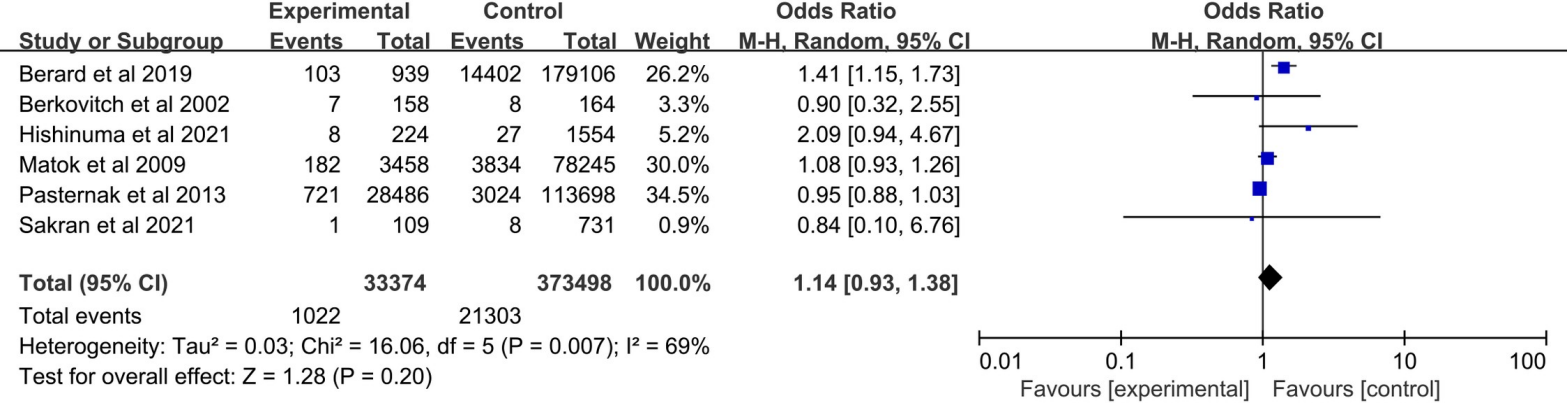
Forest plot of the association between metoclopramide use during the first trimester and the risk of major congenital malformations.

Six studies assessing a total number of 33374 metoclopramide-exposed and 373498 controls infants were included in this meta-analysis. No significant increase in the rate of major congenital malformation was detected following metoclopramide use during the first trimester of pregnancy (OR, 1.14; 95% CI, 0.93–1.38) (Fig 2).

### 3.4 Sensitivity analysis and publication bias assessment

**Fig 3:**
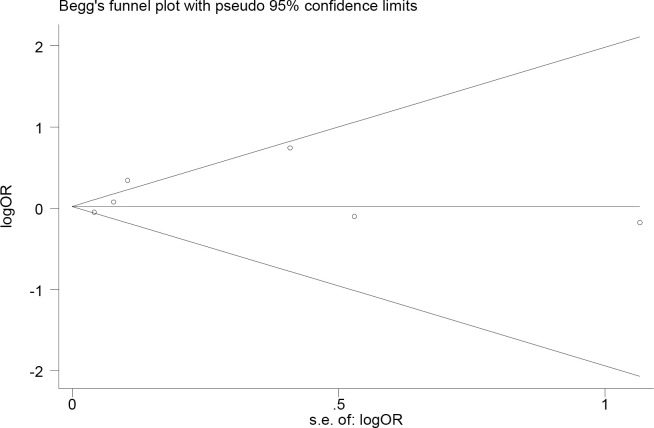
Begg’s funnel polt for publication bias. Logor, the logarithmic function values of odds ratio; s.e. of logor, the standard error of the logarithmic function values of odds ratio.

The sensitivity analysis was conducted through excluding single study from the meta-analysis at each time individually. When excluding Pasternak et al., the point estimate slightly elevated and the statistical significance (OR, 1.24; 95% CI, 1.00–1.54) altered. No significant publication bias was observed, representing as symmetrical Begg’s funnel plot (Fig 3) and a *P* value of 0.319 in Egger’s test.

## 4. Discussion

NVP is one of the most common pregnancy-related medical conditions. It would bring many negative influences to pregnant woman by generating feelings (depression, isolation, helplessness, fatigue) and hence affect their daily activities. [24–28]. Fiaschi et al [29] investigated the medical records (CPRD-GOLD) from England, finding that metoclopramide was the most commonly prescribed as second-line pharmacotherapy (11.6%) for NVP and that hospital admissions as well as antemetic prescribing increased continuously during 1998–2013. Another cross-sectional web-based study from Norwegian women indicated that metoclopramide was the second most commonly drugs used for NVP (20.8%) [30]. In the view of the widespread use of metoclopramide, its safety during pregnancy deserves our focus. This meta-analysis was based on 33374 metoclopramide-exposed and 373498 non-exposed controls and performed to investigate the association between the fetal exposure to metoclopramide during first trimester and the risk of major congenital malformation. Our results suggested that exposure to metoclopramide during the first trimester of pregnancy was not associated with the risk of major congenital malformation.

Several strengths of our meta-analysis need be mentioned as followings: 1) To the best of our knowledge, this study is the first comprehensive and the most current meta-analysis evaluating the association between metoclopramide use in the first trimester of pregnancy and the risk of major congenital malformations; 2) The studies used in the meta-analysis was selected with strict criterions about the definition of major congenital malformation, such as EUROCAT, ICD-9 and ICD-10. Besides, most studies included in our meta-analysis were retrieved case information from birth registries and medical center, which indicated that our data source is reliable; 3) This meta-analysis only included studies using metoclopramide in the first trimester when is a critical period for the development of most congenital malformation; 4) This meta-analysis only included data reporting on major congenital malformations. Data reporting minor congenital malformation were excluded because they have fewer medical, functional and societal consequences. It is worth to note that only cohort studies were included in this research, which as evidence possess higher level of credibility.

In addition to cohort study, there is a case-control study examined the association between NVP or its treatment with the most common noncardiac defects. The results showed that the use of metoclopramide in NVP patients in first trimester was not associated with Cleft Lip, Cleft Palate, Hypospadias, but the number of cases contained in this case-control study was relatively small [17]. Having included in our meta-analysis, the study of Berard et al reported that the use of metoclopramide was statistically significantly associated with an increased risk of overall major congenital malformations and that metoclopramide exposure was statistically associated with the risk of genital organ defects [8]. Although this meta-analysis did not find an association between the use of metoclopramide and the major congenital malformation, we still need to pay enough attention to the possibility that metoclopramide may cause malformation of the reproductive system. It is also deserved to arouse attention to the adverse drug reactions (ADR) of metoclopramide using during pregnancy, especially the Extrapyramidal Symptoms (EPS), which are common ADR among patients received the treatment with dopamine-receptor blocking agents. Buttino et al performed a retrospective study containing 646 pregnant women who were diagnosed as HG and received subcutaneous (s.c.) metoclopramide therapy to explore the ADR of metoclopramide [31]. As a result, 29.7% patients experienced side effects symptoms (lethargy, site irritation, agitation) and 4.6% reported EPS. The author further illustrated that the side effects were generally mild and were treated with oral or intramuscular diphenhydramine and there was no long-term sequela related to treatment with continuous s.c. metoclopramide. Tan et al performed a study to compare the effects of promethazine with those of metoclopramide for HG [32]. Results showed that promethazine and metoclopramide have similar therapeutic effects in patients, but the adverse effects (drowsiness, dizziness, and dystonia) were fewer with metoclopramide. A randomized controlled trail was conducted by Abas et al finding ondansetron and metoclopramide demonstrated similar antiemetic and antinauseant effects, but adverse effects (drowsiness, xerostomia and persistent ketonuria at 24 hours) was better with ondansetron [33]. Generally, the adverse effects of metoclopramide using during pregnancy are mild. There are also some cases reported the occurrence of EPS related to metoclopramide. Gokhale et al reported a case that a newborn baby developed dystonia after whose mother received intravenous metoclopramide as part of the pre-anesthetic medication for a lower segment (LSCS) [34]. Chua et al reported 2 cases of metoclopramide-induced acute dystonia in first trimester and speculated that it associated with the CYP2D6 poor metabolizer status [35]. Poortinga et al reported a case that a 37-year-old primigravid white woman was diagnosis as akathisia duo to the metoclopramide therapy in her 21st week of pregnancy [36].

There are several limitations in this meta-analysis should be mentioned. Several studies in this meta-analysis did not report the exact the exposure dose and duration information of metoclopramide. What’s more, some data used in this meta-analysis only contained the dispensed information of metoclopramide, but compliance of pregnant women to metoclopramide therapy were not available. All these make the specific exposure of metoclopramide possible deviation from the study data. In addition, heterogeneity was observed for the investigated outcomes, but due to the limited data, we can’t conduct stratified analyses. The sensitivity analysis evaluated the effect of omitting one study at a time from each analysis. The heterogeneity significantly declined after omission the study of Berard et al (*I*^2^ = 28%), and the OR was changed to 1.01 (95% CI, 0.89–1.15). The OR ranged from 1.14 (95% CI, 0.93–1.38) after omission of the study of Pasternak et al to 1.24 (95% CI, 1.00–1.54), and the heterogeneity was also significantly declined (*I*^2^ = 38%). Therefore, these two articles may be the reason for the high heterogeneity. However, by carefully reviewing the two full articles, we found that both of them are of high quality and strictly meet the inclusion and exclusion criteria, so they were not excluded.

In summary, this meta-analysis indicated that exposure to metoclopramide in the first trimester of pregnancy is not associated with risk of major congenital malformation.

## Supporting information

S1 File Search strategies for database. (DOC)

S2 File PRISMA 2009 checklist. (DOC)

## References

[1] Einarson TR, Piwko C, Koren G. Quantifying the global rates of nausea and vomiting of pregnancy: a meta analysis. Journal of population therapeutics and clinical pharmacology = Journal de la therapeutique des populations et de la pharmacologie clinique. 2013;20(2):e171–83. 23863575

[2] Trogstad LIS, Stoltenberg C, Magnus P, Skjaerven R, Irgens LM. Recurrence risk in hyperemesis gravidarum. Bjog-an International Journal of Obstetrics and Gynaecology. 2005;112(12):1641–5. doi: 10.1111/j.1471-0528.2005.00765.x 16305568

[3] Obstetrics Subgroup, Chinese Society of Obstetrics and Gynecology, Chinese Medical Association, Expert consensus on the diagnosis and clinical management of hyperemesis gravidarum (2015). Chinese Journal of Obstetrics Gynecology, 2015, 50(11): 801–4.

[4] Committee on Practice B-O. ACOG Practice Bulletin No. 189: Nausea And Vomiting Of Pregnancy. Obstetrics and gynecology. 2018;131(1):e15–e30. doi: 10.1097/AOG.0000000000002456 29266076

[5] Drenth JP, Engels LG. Diabetic gastroparesis. A critical reappraisal of new treatment strategies. Drugs. 1992;44(4):537–53. doi: 10.2165/00003495-199244040-00002 1281070

[6] Arvela P, Jouppila R, Kauppila A, Pakarinen A, Pelkonen O, Tuimala R. Placental transfer and hormonal effects of metoclopramide. European journal of clinical pharmacology. 1983;24(3):345–8. doi: 10.1007/BF00610052 6407846

[7] Pasternak B, Svanstrom H, Molgaard-Nielsen D, Melbye M, Hviid A. Metoclopramide in Pregnancy and Risk of Major Congenital Malformations and Fetal Death. Jama-Journal of the American Medical Association. 2013;310(15):1601–11. doi: 10.1001/jama.2013.278343 24129464

[8] Berard A, Sheehy O, Gorgui J, Zhao J-P, de Moura CS, Bernatsky S. New evidence for concern over the risk of birth defects from medications for nausea and vomitting of pregnancy. Journal of Clinical Epidemiology. 2019;116:39–48. doi: 10.1016/j.jclinepi.2019.07.014 31352006

[9] Moher D, Liberati A, Tetzlaff J, Altman DG, PRISMA Group. Preferred reporting items for systematic reviews and meta-analyses: The PRISMA statement. PLoS Medicine. 2009;6(7): e1000097. doi: 10.1371/journal.pmed.1000097 19621072 PMC2707599

[10] Wells GA, Shea B, O’connell D, Peterson J, Welch V, Losos M, et al. The Newcastle-Ottawa Scale (NOS) for assessing the quality of nonrandomised studies in meta-analyzes. Canada: The Ottawa Hospital; 2000.

[11] Stang A. Critical evaluation of the Newcastle-Ottawa scale for the assessment of the quality of nonrandomized studies in meta-analyses. European Journal of Epidemiology. 2010;25(9):603–5. doi: 10.1007/s10654-010-9491-z 20652370

[12] Congenital Anomalies–Definitions. 2020 Nov 19 [cited 28 June 2021]. In Centers for Disease Control and Prevention [Internet]. USA. Available from: https://www.cdc.gov/ncbddd/birthdefects/surveillancemanual/chapters/chapter-1/chapter1-4.html.

[13] Sun L, Xu P, Zhou YG, Zuo SR, Liu YP. Meta-analysis of polymorphism rs6311 and rs6313 in the 5-HT2AR gene and schizophrenia. Nordic Journal of Psychiatry. 2017;71(1):1–11. doi: 10.1080/08039488.2016.1217350 27598719

[14] Begg CB, Mazumdar M. Operating characteristics of a rank correlation test for publication bias. Biometrics. 1994;50(4):1088–101. 7786990

[15] Egger M, Davey Smith G, Schneider M, Minder C. Bias in meta-analysis detected by a simple, graphical test. BMJ. 1997;315(7109):629–34. doi: 10.1136/bmj.315.7109.629 9310563 PMC2127453

[16] Asker C, Wikner BN, Kallen B. Use of antiemetic drugs during pregnancy in Sweden. European Journal of Clinical Pharmacology. 2005;61(12):899–906. doi: 10.1007/s00228-005-0055-1 16328314

[17] Anderka M, Mitchell AA, Louik C, Werler MM, Hernandez-Diaz S, Rasmussen SA, et al. Medications used to treat nausea and vomiting of pregnancy and the risk of selected birth defects. Birth Defects Research Part a-Clinical and Molecular Teratology. 2012;94(1):22–30. doi: 10.1002/bdra.22865 22102545 PMC3299087

[18] Berkovitch M, Mazzota P, Greenberg R, Elbirt D, Addis A, Schuler-Faccini L, et al. Metoclopramide for nausea and vomiting of pregnancy: A prospective multicenter international study. American Journal of Perinatology. 2002;19(6):311–6. doi: 10.1055/s-2002-34469 12357422

[19] Matok I, Gorodischer R, Koren G, Sheiner E, Wiznitzer A, Levy A. The Safety of Metoclopramide Use in the First Trimester of Pregnancy. New England Journal of Medicine. 2009;360(24):2528–35. doi: 10.1056/NEJMoa0807154 19516033

[20] Sakran R, Shechtman S, Arnon J, Diav-Citrin O. Pregnancy outcome following in-utero exposure to ondansetron: A prospective comparative observational study. Reproductive Toxicology. 2021;99:9–14. doi: 10.1016/j.reprotox.2020.11.005 33212170

[21] Hishinuma K, Yamane R, Yokoo I, Arimoto T, Takahashi K, Goto M, et al. Pregnancy outcome after first trimester exposure to domperidone-An observational cohort study. The Journal of Obstetrics and Gynaecology Research. 2021;47(5):1704–10. doi: 10.1111/jog.14709 33631840 PMC8248151

[22] Marden PM, Smith DW, McDonald MJ. Congenital anomalies in the newborn infant, including minor variations. A study of 4,412 babies by surface examination for anomalies and buccal smear for sex chromatin. The Journal of pediatrics. 1964;64:357–71. doi: 10.1016/s0022-3476(64)80188-8 14130709

[23] Rasmussen SA, Olney RS, Holmes LB, Lin AE, Keppler-Noreuil KM, Moore CA, et al. Guidelines for case classification for the National Birth Defects Prevention Study. Birth Defects Research (Part A). 2003;67(3):193–201. doi: 10.1002/bdra.10012 12797461

[24] Smith C, Crowther C, Beilby J, Dandeaux J. The impact of nausea and vomiting on women: a burden of early pregnancy. Australian & New Zealand Journal of Obstetrics & Gynaecology. 2000;40(4):397–401. doi: 10.1111/j.1479-828x.2000.tb01167.x 11194422

[25] Attard CL, Kohli MA, Coleman S, Bradley C, Hux M, Atanackovic G, et al. The burden of illness of severe nausea and vomiting of pregnancy in the United States. American Journal of Obstetrics and Gynecology. 2002;186(5):S220–S7. doi: 10.1067/mob.2002.122605 12011890

[26] Mazzotta P, Stewart D, Atanackovic G, Koren G, Magee LA. Psychosocial morbidity among women with nausea and vomiting of pregnancy: prevalence and association with anti-emetic therapy. Journal of Psychosomatic Obstetrics & Gynecology. 2000;21(3):129–36. doi: 10.3109/01674820009075620 11076334

[27] O’Brien B, Evans M, White-McDonald E. Isolation from "being alive": Coping with severe nausea and vomiting of pregnancy. Nursing Research. 2002;51(5):302–8. doi: 10.1097/00006199-200209000-00006 12352778

[28] Chou FH, Lin LL, Cooney AT, Walker LO, Riggs MW. Psychosocial factors related to nausea, vomiting, and fatigue in early pregnancy. Journal of Nursing Scholarship. 2003;35(2):119–25. doi: 10.1111/j.1547-5069.2003.00119.x 12854291

[29] Fiaschi L, Nelson-Piercy C, Deb S, King R, Tata LJ. Clinical management of nausea and vomiting in pregnancy and hyperemesis gravidarum across primary and secondary care: a population-based study. Bjog-an International Journal of Obstetrics and Gynaecology. 2019;126(10):1201–11. doi: 10.1111/1471-0528.15662 30786126

[30] Heitmann K, Solheimsnes A, Havnen GC, Nordeng H, Holst L. Treatment of nausea and vomiting during pregnancy -a cross-sectional study among 712 Norwegian women. European Journal of Clinical Pharmacology. 2016;72(5):593–604. doi: 10.1007/s00228-016-2012-6 26815908

[31] Buttino L Jr, Coleman SK, Bergauer NK, Gambon C, Stanziano GJ. Home subcutaneous metoclopramide therapy for hyperemesis gravidarum. Journal of Perinatology. 2000;20(6):359–62. doi: 10.1038/sj.jp.7200398 11002874

[32] Tan PC, Khine PP, Vallikkannu N, Omar SZ. Promethazine compared with metoclopramide for hyperemesis gravidarum: a randomized controlled trial. The American College of Obstetricians and Gynecologists. 2010;115(5):975–81. doi: 10.1097/AOG.0b013e3181d99290 20410771

[33] Abas MN, Tan PC, Azmi N, Omar SZ. Ondansetron compared with metoclopramide for hyperemesis gravidarum: a randomized controlled trial. The American College of Obstetricians and Gynecologists. 2014;123(6):1272–9. doi: 10.1097/AOG.0000000000000242 24807340

[34] Gokhale SG, Panchakshari MB. Maternal medication causing drug dystonia in a newborn: placental transfer of drugs. The Journal of Maternal-Fetal and Neonatal Medicine. 2004;16(4):215–7. doi: 10.1080/14767050400014436 15590449

[35] Chua EW, Harger SP, Kennedy MA. Metoclopramide-Induced Acute Dystonic Reactions May Be Associated With the CYP2D6 Poor Metabolizer Status and Pregnancy-Related Hormonal Changes. Frontiers in Pharmacology. 2019;22(10):931. doi: 10.3389/fphar.2019.00931 31507424 PMC6713716

[36] Poortinga E, Rosenthal D, Bagri S. Metoclopramide-induced akathisia during the second trimester of a 37-year-old woman’s first pregnancy. Psychosomatics. 2001;42(2):153–6. doi: 10.1176/appi.psy.42.2.153 11239130

