# Supplementary figures and images for: Use of metoclopramide in the first trimester and risk of major congenital malformations: A systematic review and meta-analysis
Source: PLoS One. 2021 Sep 20;16(9):e0257584. doi: 10.1371/journal.pone.0257584 (PMC8452057; doi:10.1371/journal.pone.0257584)

**S1 File. Search strategies for database**

**Pubmed:**


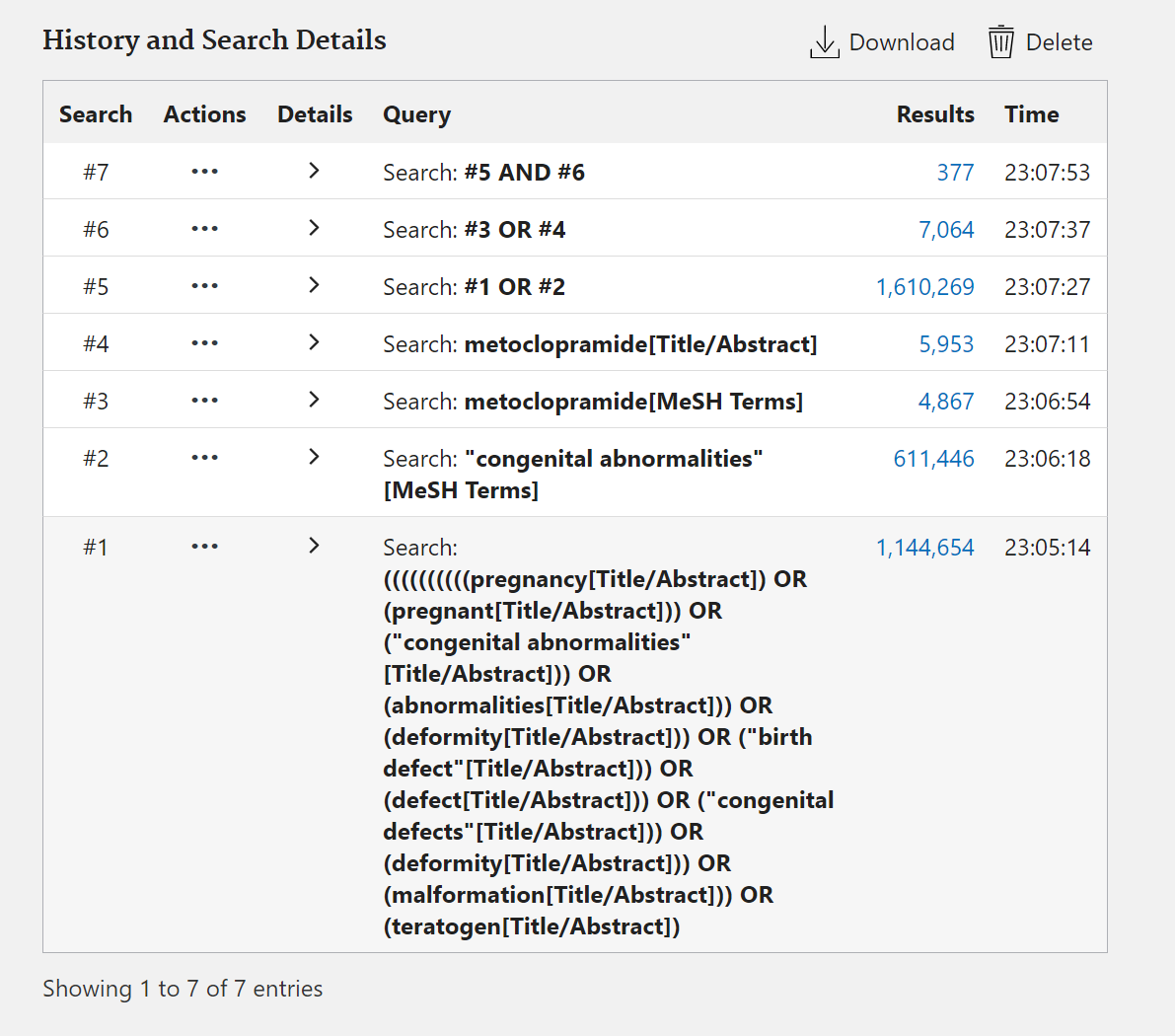


**Embase:**


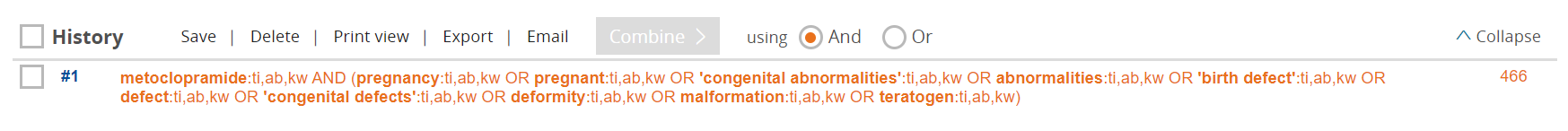


**Web of science:**


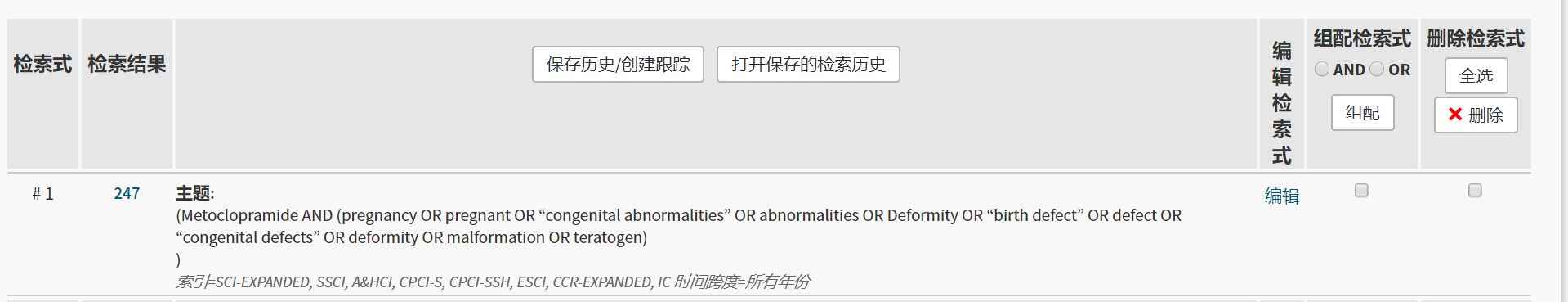


**Cochrane library:**


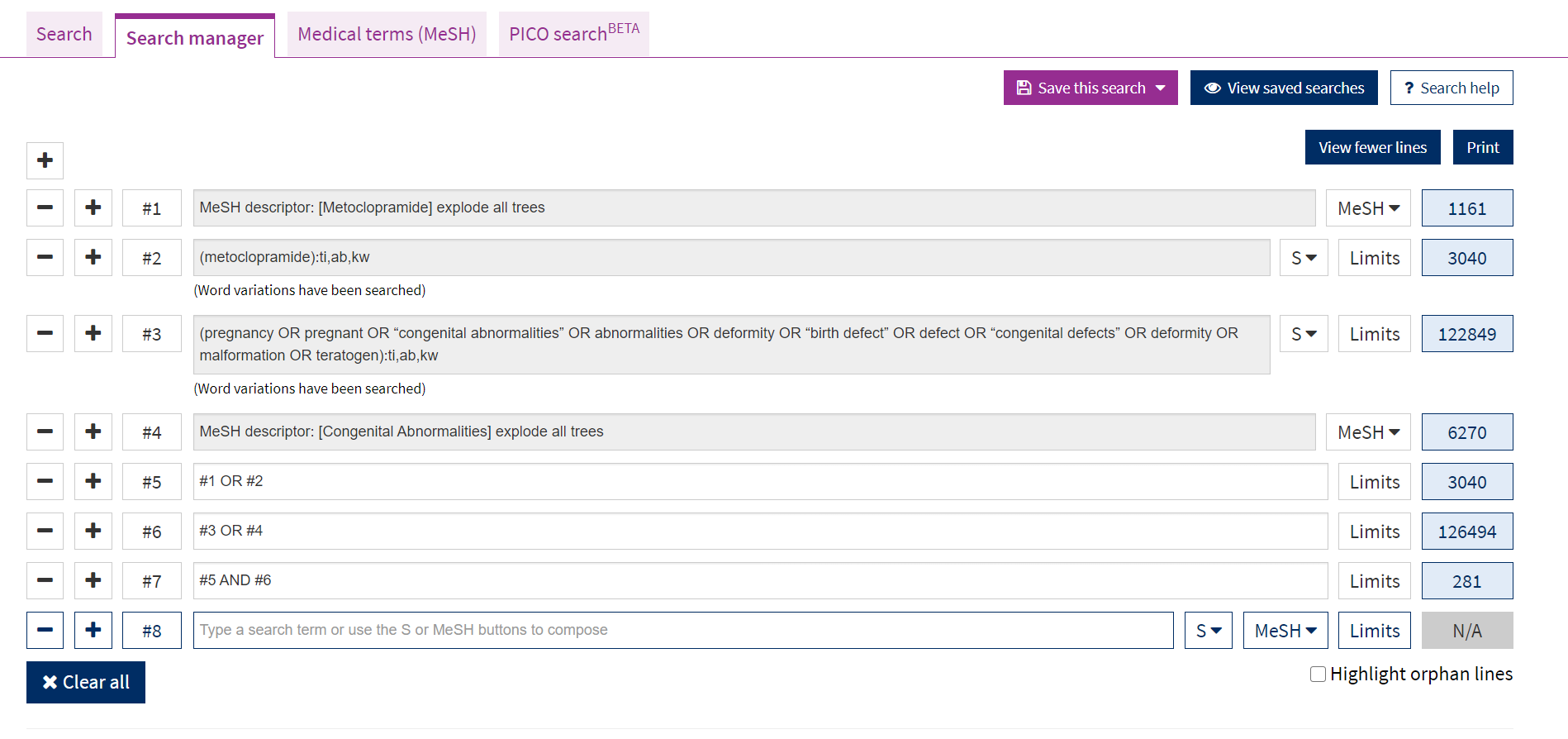

Supplement: S1 File — (DOC) [file pone.0257584.s001.doc]
